# Molecular genetic study on *GATA5* gene promoter in acute myocardial infarction

**DOI:** 10.1371/journal.pone.0248203

**Published:** 2021-03-08

**Authors:** Zhipeng Song, Lu Chen, Shuchao Pang, Bo Yan

**Affiliations:** 1 Department of Medicine, Shandong University School of Medicine, Jinan, Shandong, China; 2 Center for Molecular Medicine, Yanzhou People’s Hospital, Affiliated Hospital of Jining Medical University, Jining Medical University, Jining, Shandong, China; 3 Shandong Provincial Key Laboratory of Cardiac Disease Diagnosis and Treatment, Affiliated Hospital of Jining Medical University, Jining Medical University, Jining, Shandong, China; 4 Shandong Provincial Sino-US Cooperation Research Center for Translational Medicine, Affiliated Hospital of Jining Medical University, Jining Medical University, Jining, Shandong, China; University of Tampere, FINLAND

## Abstract

**Background:**

Acute myocardial infarction (AMI) is a severe type of coronary artery disease, caused by coronary occlusion and followed by cardiac ischaemia. GATA binding protein 5 (GATA5) is an important member of GATA family and plays an important role in vascular inflammation, endothelial function, oxidative stress and cell metabolism. Previous studies have shown that the DNA sequence variants (DSVs) in *GATA4* and *GATA6* promoter can increase susceptibility to AMI. In this study, we explored the relationship between *GATA5* promoter and AMI for the first time, hoping to provide a new genetic basis for understanding the pathogenesis of AMI.

**Methods:**

*GATA5* promoter was sequenced in 683 individuals (332 AMI patients and 351 controls). The transcriptional activity of the *GATA5* promoter with or without DSVs in HEK-293 cells, H9c2 cells and primary neonatal rat cardiomyocytes were examined by Promega Dual-Luciferase® Reporter Assay system. Electrophoretic mobility shift assay (EMSA) was performed to explore whether the DSVs interfered with the binding of transcription factors (TFs).

**Results:**

Nine mutations have been found in *GATA5* promoter, eight of them evidently altered the transcriptional activity of the *GATA5* promoter, five of them disrupted the binding of TFs (such as farnesoid X receptor). Furthermore, haplotype AT (across rs80197101 and rs77067995) is a dangerous haplotype of AMI. Genotype GA and allele A of rs80197101 and genotype CT and allele T of rs77067995 are the risk factors of AMI.

**Conclusions:**

DSVs in *GATA5* promoter can increase susceptibility to AMI. But the mechanism remains to be verified in *vivo*.

## 1. Introduction

Cardiovascular diseases (CVDs) are the leading cause of death globally [1]. Acute myocardial infarction (AMI) is a serious type of CVDs, mainly related to coronary occlusion, accounting for more than 20% of cardiovascular deaths, and the 5-year survival rate of AMI is only 55% [2]. Previous studies have confirmed that heritability of AMI is 50% to 60% [3]. But, the genetic cause and potential molecular mechanism of AMI are still unclear [4].

GATA binding protein 5 (GATA5) is a member of GATA family and plays an important role in CVDs as *GATA5* loss-of-function mutations are closely related to congenital heart disease, atrial fibrillation, hypertension [5–8]. The latest epidemiology shows that CVD is becoming increasingly common in the elderly Adult Congenital Heart Disease population [9]. The incidence of AMI in patients with congenital heart disease is significantly higher than that in healthy controls [10–12]. Furthermore, GATA5 can regulate cell metabolism [13, 14], coronary artery development [15–17], vascular inflammation, oxidative stress and endothelial function [7, 18], by affecting the expression of bone morphogenic protein-4 (BMP4), Amp-activated protein kinase (AMPK), Friend of GATA-2 (FOG-2) and other cytokines. Therefore, the imbalance of the expression level of *GATA5* gene may increase susceptibility to AMI.

Previous studies have shown that the DNA sequence variations (DSVs) of *GATA4* and *GATA6* gene promoter can increase susceptibility to AMI [19, 20]. In this study, we investigated the correlation between DSVs of *GATA5* gene promoter and AMI for the first time, which is expected to provide a new genetic basis for the prevention, diagnosis and treatment of AMI.

## 2. Materials and methods

### 2.1 Subjects

332 AMI patients and 351 ethnic-matched healthy controls were respectively recruited from Cardiac Care Unit and Physical Examination Center, Affiliated Hospital of Jining Medical University, Jining Medical University, Jining, Shandong, China.

The research was approved by the Human Ethic Committee of the Affiliated Hospital of Jining Medical University (2018-FY-070) and was carried out according to the principles of the Declaration of Helsinki. Written informed consents were obtained from all participants.

### 2.2 Isolation of primary neonatal rat cardiomyocytes (NRCMs)

According to the literature [21, 22], Method of isolating primary NRCMs have been improved. Retrieve neonatal rat pups (1–3 days old, gender unknown) from the mother. After disinfecting the pups with 75% alcohol, their hearts were removed quickly. The non-cardiac tissues and residual blood were carefully removed in PBS containing 100 U·mL^-1^ penicillin-streptomycin before shredded the hearts. Digest the chopped tissues with 0.25% trypsin for 3–4 minutes, then discard the supernatant. Repeat digestion until the tissues are digested completely, supernatants were retained and transferred into the termination medium (15% FBS and 100 U·mL^-1^ penicillin-streptomycin) to terminate digestion. The supernatants filtered by 70μm cell strainer were centrifuged (1000 rpm, 10 min) in a new EP tube. Collected cells were resuspended with DMEM (10% FBS and 100 U·mL^-1^ penicillin-streptomycin) and seeded in the petri dish, cultured at 37°C in a 5% CO_2_ incubator for 1.5 hours. The upper culture medium rich in primary NRCMs was carefully collected and inoculated to a six-well plate. The medium was replaced with fresh medium 24 hours later, and primary NRCMs were transfected 36–48 hours later.

### 2.3 Direct DNA sequencing

Fasting peripheral blood (3 ml) was collected from AMI patients before treatment and from healthy subjects during physical examination. Genomic DNAs were extracted from leukocytes according to the instructions of QIA amp DNA Mini kit (Qiagen, Inc., Valencia, CA, USA). According to the human *GATA5* gene promoter sequence (NCBI, NC_000020.11) in NCBI, the transcription start site (TSS) of *GATA5* gene promoter was located at g.62475521 (+1). The DNA fragment (836 bp, from -1234 bp to -399 bp to the TSS) was selected and was obtained by polymerase chain reaction (PCR) with TaKaRa LA Taq® with GC Buffer (Takara Biomedical Technology (Beijing) Co., Ltd. Code No.: RR02BG). PCR primers of *GATA5* gene promoter sequence were designed by Primer Premier 5.0 software and then synthesized by Sangon Biotech Co., Ltd. (Shanghai, China), (GATA5-F: 5´-AGTGCGAGCGGGACACGGTT-3´; GATA5-R: 5´-GAGCACTCACCAGCGGGCAG-3´). Thermocycling conditions were as follows: template DNA 2.5 μl, upstream/downstream primers (50 μmol/L) 0.25 μl, Taq enzyme 0.5 μl, dNTP MIX 8 μl, GC buffer I 25 μl, DMSO 2.5 μl, ddH2O 11 μl; a total of 35 cycles of denaturing at 94°C for 30 sec, annealing at 62°C for 50 sec, extending at 72°C for 40 sec. PCR products were sequenced on the 3730 DNA Analyzer (Applied Biosystems, Foster City, CA, USA). The DNA fragment sequences were then compared with normal *GATA5* gene promoter sequence with DNAMAN program (version 5.2.2; Lynnon BioSoft, Quebec, Canada). The effects of the DSVs in *GATA5* gene promoter on binding sites for transcription factors (TFs) were predicted by Transfac program (https://portal.genexplain.com/).

### 2.4 Analysis of transcriptional activity of *GATA5* gene promoter

In order to generate expression vectors, the Hind III and Kpn I sites of pGL3-basic (Promega Corporation) were inserted into the variant and wild type DNA fragments (836bp, from -1234 bp to -399 bp) of *GATA5* gene promoters by PCR using the primers [Forward: 5´-(Kpn I) -AGTGCGAGCGGGACACGGTT-3´; Reverse: 5´-(Hind III)-GAGCACTCACCAGCGGGCAG -3´]. The linked single nucleotide polymorphisms (SNPs) [g.62476317G>A (rs80197101) and g.62476223C>T (rs77067995)] were constructed as one expression vector (pGL3-62476317A+62476223T). Designated expression constructs (pGL3-DNA fragments of interesting) expressed firefly luciferase activity, and pRL-TK (25 ng) expressed renilla luciferase. The pRL-TK and empty vector (pGL3-basic) were used as internal and negative control, respectively.

These expression vectors were transfected into H9c2 cells [rat cardiomyocyte line; CRL-1446; American Type Culture Collection (ATCC)], HEK-293 cells (CRL-1573; ATCC, Manassas, VA, USA) and primary NRCMs which have been widely used in transient transfection experiments. Cells were seeded in 6-well plates and grown to 70% to 80% confluence, and then designated expression constructs (1.0 μg) were used to transfect the cells in each well. Forty-eight hours later, dual-luciferase activities of the transfected cells were examined using the Promega Dual-Luciferase® Reporter Assay system on a Promega Glomax 20/20 luminometer (both Promega Corporation). The transcriptional activity of the *GATA5* gene promoter was expressed as the ratio of firefly luciferase activity over renilla luciferase activity. Transcriptional activity of wild type *GATA5* gene promoter was designed as 100%, relative transcriptional activities of the variant *GATA5* gene promoters were calculated.

### 2.5 Electrophoretic mobility shift assay (EMSA)

EMSA was carried out using the LightShift® Chemiluminescent EMSA kit (Thermo Fisher Scientific, Inc.) according to the manufacturer’s protocol. NE-PER® Nuclear and Cytoplasmic Extraction Reagent kit (Thermo Fisher Scientific, Inc.) was used to prepare nuclear extracts from H9c2 and HEK-293 cells, the concentrations of nuclear extract were measured by Bradford protein assay. Double-stranded oligonucleotide fragments (30 bp) containing DSVs were biotinylated and used as probes (Table 1). Nuclear extracts (3.0 μg) and probes (0.2 pmol) were incubated for 20 min at room temperature, separated on a native 6% polyacrylamide gel at 100 V for 90 min, and then transferred onto a nylon membrane (Thermo Fisher Scientific, Inc.) at 380 mA for 30 min. UV Stratalinker 1800 (Stratagene, Santa Clara, CA, USA) was used to cross-link oligonucleotides onto the nylon membrane, and the LightShift® Chemiluminescent EMSA kit (Thermo Fisher Scientific, Inc.) was used for chemiluminescence detection.

**Table 1:** The double-stranded biotinylated oligonucleotides for the EMSA.

DSVs	Oligonucleotide sequences	Locations
g.62476323-24 GG>AA	TTTAGGCCAGCCTTC(GG/AA)CGGGGGCCGGGGCA	g.62476309-g.62476339
g.62476197 G>A	ACTGGTCCGGGCTCC(G/A)CGCTGGCCGCCCCG	g.62476183-g.62476212
g.62476171 C>T (rs1341970027)	CCCGTGTCGTGCGTC(C/T)TTGTCGCCAAGCCC	g.62476157-g.62476186
g.62476123 A>G (rs1435326263)	GGGTCACGTGGCTCT(A/G)CGGCCGGAGCCCCA	g.62476109-g.62476138
g.62476046 G>A	ACTGGTCCGGGCTCC(G/A)CGCTGGCCGCCCCG	g.62476032-g.62476061
g.62475977 C>G (rs1294169077)	CTTCGGCCCGCCGGT(C/G)GCCGACCCACCGCC	g.62475963-g.62475992
g.62476317 G>A (rs80197101)	CAGCCTTCGGCGGGG(G/A)CCGGGGCAGGGAGG	g.62476303-g.62476332
g.62476223 C>T (rs77067995)	AGCCCTCGGCCCGCC(C/T)GTCTAGCTGCAATG	g.62476209-g.62476238

### 2.6 Statistical analysis

Results of transient transfection were independently repeated at least three times and verified by others, the value was expressed as the mean ± standard error of the mean and compared by a standard Student’s t-test. Statistical analysis was carried out by SPSS software version 19.0 (IBM Corporation, USA), the measurement data were expressed as mean ± standard deviation. For SNPs (rs80197101, rs145936691 and rs77067995), Hardy-Weinberg equilibrium was tested using a goodness-of-fit Chi-square, and R×C chi-square test was used to analyze the distribution difference of genotype and allele frequency between AMI and control group. The correlation between SNPs and AMI was analyzed by logistic regression analysis, which was expressed by odds ratio (OR) and 95% confidence interval (CI). The linkage disequilibrium of the SNPs was analyzed by Haploview 4.2 software. Haplotypes were analyzed by SHEsis online software (http://analysis.bio-x.cn/myAnalysis.php). The statistical power was analyzed by Genetic Association Study Power Calculator (http://csg.sph.umich.edu/abecasis/gas_power_calculator/index. html). All statistical tests were two-sided and P<0.05 was considered statistically significant.

## 3. Results

### 3.1 Analysis of clinical characteristics in AMI and control group

The proportion of male, smoking, hypertension (HTN), diabetes mellitus (DM) and the level of age in AMI group were significantly higher than that in control group (*P* = 0.012, *P*<0.001, *P*<0.001, *P*<0.001, *P*<0.001). The level of high density lipoprotein cholesterol (HDL-C) in the control group was significantly higher than that in AMI group (*P*<0.001). Due to the use of lipid-lowering drugs in AMI patients, the levels of body mass index (BMI), low density lipoprotein cholesterol (LDL-C) and total cholesterol (TC) in the control group were significantly higher than those in AMI group (*P* = 0.010, *P*<0.001, *P*<0.001). The levels of systolic blood pressure (SBP), diastolic blood pressure (DBP) and triglyceride (TG) was no significant difference between the two groups (*P* = 0.219, *P* = 0.825, *P* = 0.650) (Table 2). In the follow-up analysis, we corrected the above risk factors.

**Table 2:** Analysis of the clinical characteristics.

Parameters	Controls (n = 351)	AMI cases (n = 332)	P
Age, mean (SD), years	45.25 (13.46)	63.41 (13.17)	<0.001
Male/female (n)	221/130	239/93	0.012
Smoking [n (%)]	57 (16.2)	173 (52.1)	<0.001
HTN [n (%)]	86 (24.5)	147 (44.3)	<0.001
DM [n (%)]	26 (7.4)	73 (22.0)	<0.001
BMI, mean (SD), kg/m^2^	25.57 (3.70)	24.77 (3.74)	0.010
SBP, mean (SD), mmHg	127.68 (17.68)	125.62 (22.90)	0.219
DBP, mean (SD), mmHg	78.54 (12.46)	78.29 (15.32)	0.825
HDL-C, mean (SD), mmol/L	1.32 (0.30)	1.05 (0.37)	<0.001
LDL-C, mean (SD), mmol/L	2.80 (0.72)	2.52 (0.80)	<0.001
TG, mean (SD), mmol/L	1.44 (1.08)	1.48 (0.94)	0.650
TC, mean (SD), mmol/L	4.94 (1.44)	4.27 (1.06)	<0.001

HTN, hypertension; DM, Diabetes Mellitus; BMI, body mass index; SBP, Systolic blood pressure; DBP, Diastolic blood pressure; HDL-C, High density Lipoprotein cholesterol; LDL-C, Low Density Lipoprotein cholesterol; TG, Triglyceride; TC, Total Cholesterol. Quantitative data including age, BMI, SBP, DBP, HDL-C, LDL-C, TG and TC was expressed as Mean±Standard Deviation.

### 3.2 The identified DSVs in the *GATA5* gene promoter

**Fig 1:**
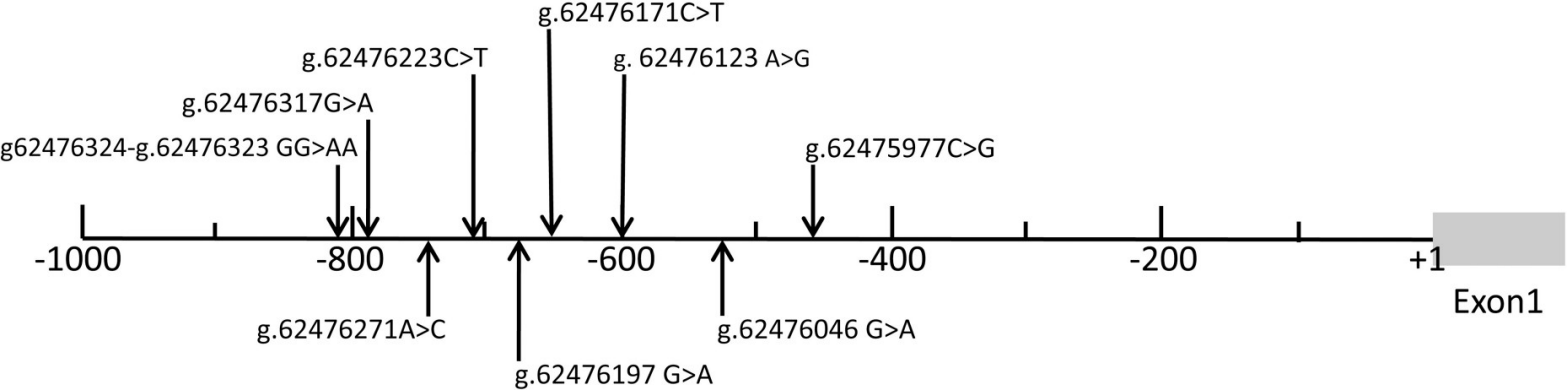
Locations of the mutational sites in *GATA5* gene promoter in all 683 individuals (332 AMI patients and 351 controls). The transcription start site is at the position of g.62475521 (+1) in the first exon. The numbers represents the genomic DNA sequences of the *GATA5* gene (NC 000020.11.).

**Fig 2:**
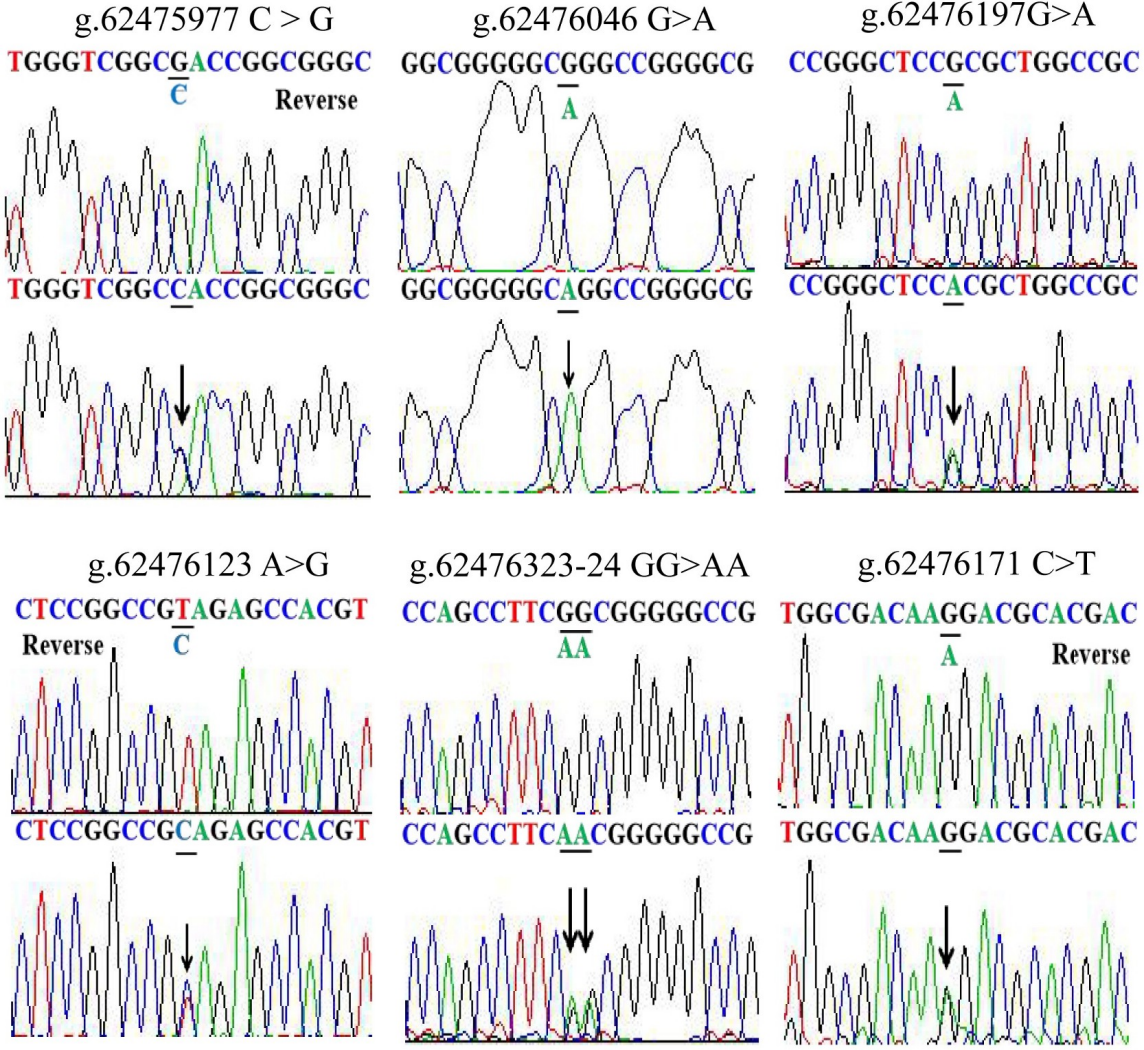
Sequencing chromatograms of the DSVs only identified in 332 AMI patients. Sequence orientations of the DSVs are marked, top panels show wild type and bottom panels represent heterozygous variants. DSVs are marked with arrows. Heterozygous variant means that only one of a pair of alleles has a mutation.

**Fig 3:**
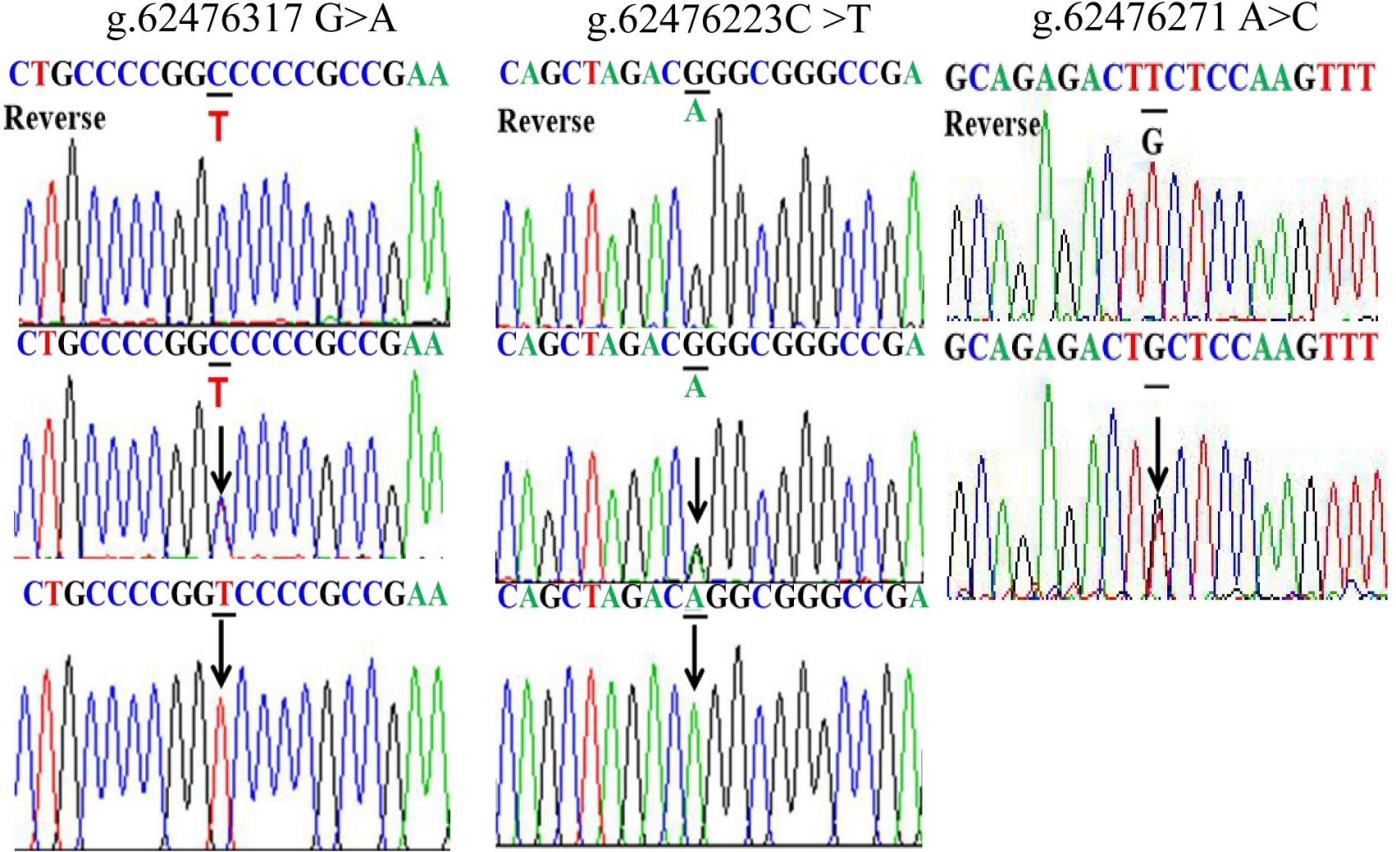
Sequencing chromatograms of the DSVs found in both 332 AMI patients and 351 controls. Sequence orientations are marked, top panel shows wild type, middle panel shows heterozygous variants and bottom panel shows homozygous variants. DSVs are marked with arrows. Homozygous variant means that there are mutations in both alleles. Heterozygous variant means that only one of a pair of alleles has a mutation.

The *GATA5* promoter was sequenced in all 683 individuals and nine DSVs were identified. We re-sequenced these nine DSVs to rule out the possibility that some DSVs arose due to PCR mistakes. The frequencies and locations of these nine DSVs are summarized in Table 3 and depicted in Fig 1. The sequencing chromatograms of them are shown in Figs 2 and 3. Three novel DSVs (g.62476323-24GG>AA, g.62476197G>A and g.62476046G>A) and three SNPs [g.62476171C>T (rs1341970027), g.62476123A>G (rs1435326263) and g.62475977C>G (rs1294169077)] were only identified in AMI patients (Fig 2). The other three SNPs [g.62476317G>A (rs80197101), g.62476223C>T (rs77067995) and g.62476271A>C (rs145936691)] were found in both AMI patients and controls (Fig 3). Of note, both g.62476317G>A (rs80197101) and g.62476223C>T (rs77067995) were identified in forty-two AMI patients and twenty-five healthy controls (*X*^2^ = 6.459, *P* = 0.040; *X*^2^ = 6.459, *P* = 0.040, respectively), while g.62476271 A>C (rs145936691) was reported in controls and AMI patients with similar frequencies (*P* = 0.722).

**Table 3:** DSVs of GATA5 gene promoter in AMI patients and controls.

DSVs	Genotypes	Location^a^	Controls (n = 351)	AMI cases (n = 332)	P
g.62476323-24 GG>AA	GG/AA	-803	0	1	--
g.62476317 G>A ( rs80197101)	GA	-796	25	41	0.040
	AA		0	1	
g.62476271 A>C (rs145936691)	AC	-750	9	10	0.722
g.62476223 C >T (rs77067995)	CT	-702	25	41	0.040
	TT		0	1	
g.62476197 G>A	GA	-676	0	1	--
g.62476171 C>T (rs1341970027)	CT	-650	0	1	--
g 62476123 A>G (rs1435326263)	AG	-602	0	1	--
g.62476046 G>A	AA	-525	0	1	--
g.62475977 C>G (rs1294169077)	CG	-456	0	1	--

^a^, DSVs are located upstream (-) to the transcription start site of GATA5 gene at g.62475521 (+1) of NC_000020.11.

### 3.3 Hardy-Weinberg equilibrium test and analysis of genotype and allele frequencies

The results of Hardy-Weinberg equilibrium test showed that rs80197101, rs145936691 and rs77067995 were in accordance with Hardy-Weinberg equilibrium (For case: *P* = 0.722, *P* = 0.781, *P* = 0.722; for control: *P* = 0.489, *P* = 0.808, *P* = 0.489), indicating that the samples had good representativeness and came from the population that was genetic equilibrium.

For rs80197101, there was significant difference in the distribution of rs80197101 genotypes between AMI and control group (*X*^*2*^ = 6.459, *P* = 0.040), the frequencies of GA+AA genotypes and A allele in AMI group were significantly higher than that in control group (*X*^*2*^ = 5.893, *P* = 0.015; *X*^*2*^ = 6.128, *P* = 0.013). The frequency distribution of alleles and genotypes of rs77067995 between AMI and control group was the same as that of rs80197101. For rs145936691, the frequency distribution of alleles and genotypes between AMI and control group was not significantly different (*X*^*2*^ = 0.127, *P* = 0.722; *X*^*2*^ = 0.125, *P* = 0.724).

### 3.4 Logistic regression, linkage disequilibrium and haplotype analysis

Correlation between SNPs (rs80197101 and rs77067995) and AMI was analyzed by Logistic regression. The results showed that GA and GA+AA genotypes of rs80197101 locus were correlated with the occurrence of AMI (OR = 1.844, 95% CI: 1.094~3.107, *P* = 0.022; OR = 1.889, 95% CI: 1.123~3.176, *P* = 0.017). After adjusting for age, gender, smoking, HTN, DM, HDL-C, LDL-C, TG and TC, the GA and GA+AA genotypes of rs80197101 locus were still correlated with the occurrence of AMI (OR = 2.263, 95%CI: 1.009~5.079, *P* = 0.048; OR = 2.297, 95%CI: 1.028~5.135, *P* = 0.043). As for the effect to result in AMI, the GA and GA+AA genotypes were 2.263 and 2.297 times higher than that of GG genotype, respectively. The correlation between rs77067995 and AMI was the same as that of rs80197101.

Rs80197101 and rs77067995 showed perfect linkage disequilibrium (*D´* = 1.000, *r*^*2*^ = 1.000). The haplotypes composed of these two SNPs were analyzed (frequency<0.03 was ignored in the analysis), and found that rs80197101G>A and rs77067995C>T formed two haplotypes (AT and GC). Haplotype AT exhibited higher frequencies in AMI group than in control group (OR = 1.875, 95%CI: 1.132–3.106, *P* = 0.013), while haplotype GC exhibited higher frequencies in control group than in AMI groups (OR = 0.533, 95%CI: 0.322~0.884, *P* = 0.013). Considering the two haplotypes (AT and GC) together, the difference of frequency distribution between the two groups was also statistically significant (*P* = 0.013).

### 3.5 A power statistical calculation in this case-control study

Genetic Association Study Power Calculator (http://csg.sph.umich.edu/abecasis/gas_power_calculator/index.html) was used to calculate the statistical power under these situations: the ratio of cases (n = 330) to controls (n = 350) was 0.943, significance level was 0.05, disease model was multiplicative, disease prevalence was 0.10, disease allele frequency was 0.2338 (159/680), genotype relative risk was 1.4. Finally, the values of statistical power was 0.863.

### 3.6 Putative binding sites for TFs affected by DSVs

Transfac program (https://portal.genexplain.com/) was used to predict whether and which putative binding sites for TFs were affected by DSVs. The DSV g.62476323-24 GG>AA abolished the binding site for E2F related factors (cttC**GG**CGgggg) and AP-2 (ccagccttC**GG**CGgg). The DSV g.62476197G>A modified the binding sites for ZFP161 (tcC**G**CGCtggccgc). The SNP g.62475977C>G (rs1294169077) abolished the binding site for farnesoid X receptor (FXR) (cccgccGGT**C**Gccgaccca). The SNP g.62476317G>A (rs80197101) abolished the binding site for Kruppel-like factor 6 (KLF6) (gcGGGG**G**). The SNP g.62476223C>T (rs77067995) created the binding site for Small mothers against decapentaplegic (SMAD) (cGCC**T**Gt). The SNP g.62476171C>T (rs1341970027) abolished the binding site for LRH-1(cgtC**C**TTGtcg) and Steroidogenic factor-1 (SF-1) (cgtC**C**TTGt) group, created the binding sites for transcription factor 7 (TCF7) related factors (tC**T**TTGt). The SNP g.62476123A>G (rs1435326263) created the binding sites for BEN (CT**G**CGgcc). The DSV g.62476046G>A modified the binding sites for AP-2 (cgcggcggGGGC**G**gg) and Krüppel-like factor 4 (KLF4) (ggGGCG**G**).

Theses TFs were involved in a variety of biological processes such as DNA repair, DNA replication, differentiation, proliferation, apoptosis, anti-inflammatory activity and lipid metabolism [23–32].

### 3.7 Analysis of transcriptional activity of *GATA5* gene promoter

Whether in HEK-293 cells, H9c2 cells or primary NRCMs, transcriptional activity of the wild-type *GATA5* gene promoter (pGL3-WT) was designed as 100%. According to our results, the transcriptional activity of the promoter-free vector (pGL3-basic) was close to zero compared with pGL3-WT, indicating that the transcriptional activity of other expression vectors we constructed were credible.

**Fig 4:**
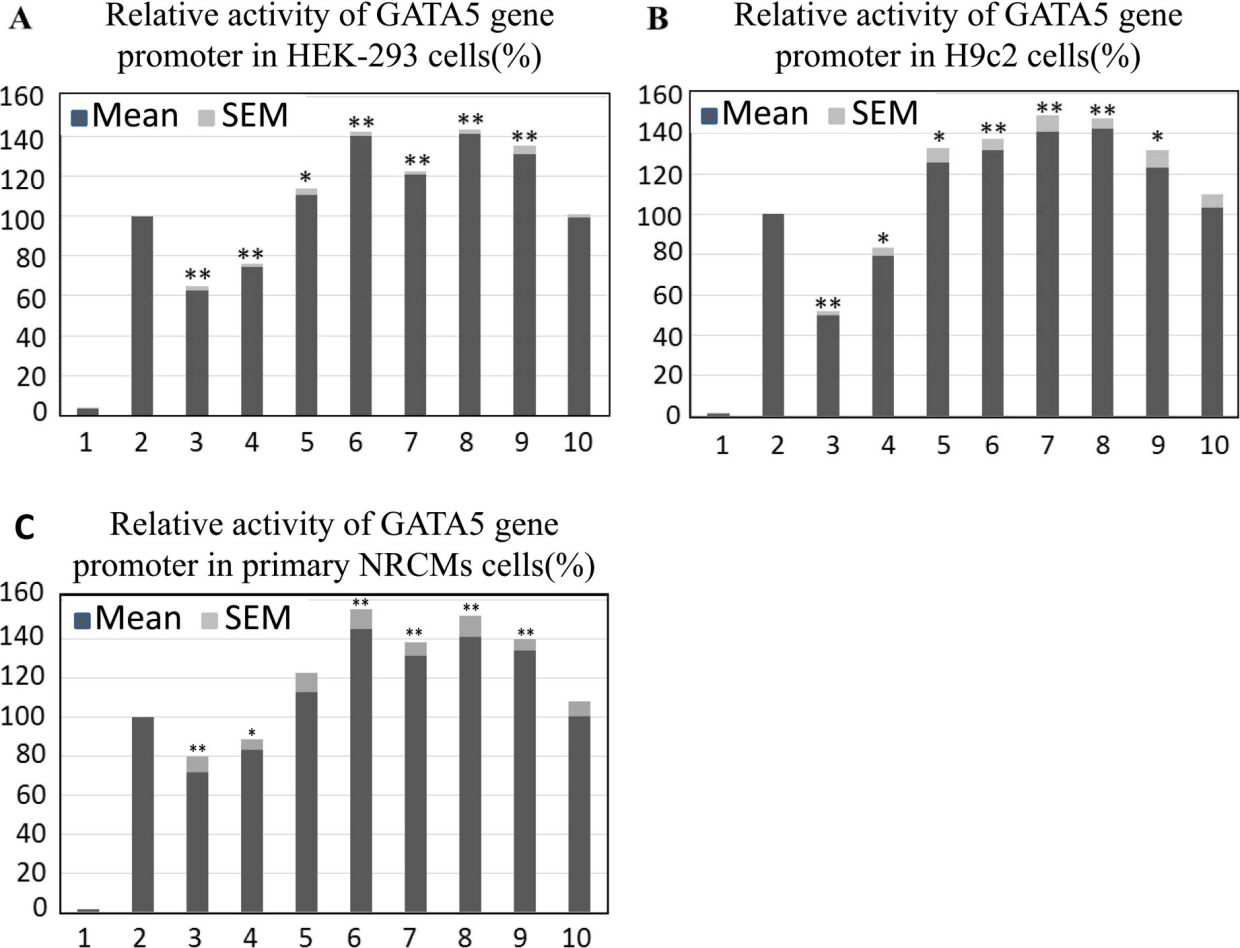
Relative activities of wild type and variant *GATA5* gene promoters in HEK-293 (A), H9c2 (B) and primary NRCMs (C) cells. Wild type and variant *GATA5* gene promoters were cloned into reporter gene vector pGL3 and transfected into HEK-293, H9c2 and primary NRCMs cells. The transfected cells were collected and dual-luciferase activities were assayed. Empty vector pGL3-basic is used as a negative control. Transcriptional activity of the wild type *GATA5* gene promoter was designed as 100%. Relative activities of *GATA5* gene promoters were calculated. The transfection results were independently repeated at least three times. Lanes 1, pGL3-basic; 2, pGL3-WT; 3, pGL3-62475977G; 4, pGL3-62476046A; 5, pGL3-62476197A; 6, pGL3-62476123G; 7, pGL3- 62476323-24AA; 8, pGL3-62476317A+62476223T; 9, pGL3-g.62476171T; 10, pGL3- 62476271C. NRCMs, neonatal rat cardiomyocytes; SEM, Standard Error of the Mean; WT, wild type. *, P<0.05; **, P<0.01.

In HEK-293 cells, pGL3-62475977G and pGL3-62476046A (identified only in AMI patients) evidently repressed the transcriptional activity of *GATA5* gene promoter (*P*<0.01), pGL3-62476197A, pGL3-62476123G, pGL3-62476323-24AA and pGL3-g.62476171T (identified only in AMI patients) and pGL3-62476317A+62476223T (identified in both AMI patients and controls, but the frequencies in AMI group were significantly higher than that in healthy control group, *P* = 0.040) evidently increased the transcriptional activity of *GATA5* gene promoter (*P*<0.05). As expected, pGL3- 62476271C (identified in both AMI patients and controls with similar frequencies, *P* = 0.722) did not affect the activity of *GATA5* gene promoter significantly(*P*>0.05; Fig 4A).

The transfection results of H9c2 cells (Fig 4B) and primary NRCMs (Fig 4C) were consistent with those of HEK-293 cells, indicating that the effect of the above mutation sites on the transcriptional activity of *GATA5* gene promoter was not tissue-specific.

### 3.8 The binding for TFs interfered by the DSVs

**Fig 5:**
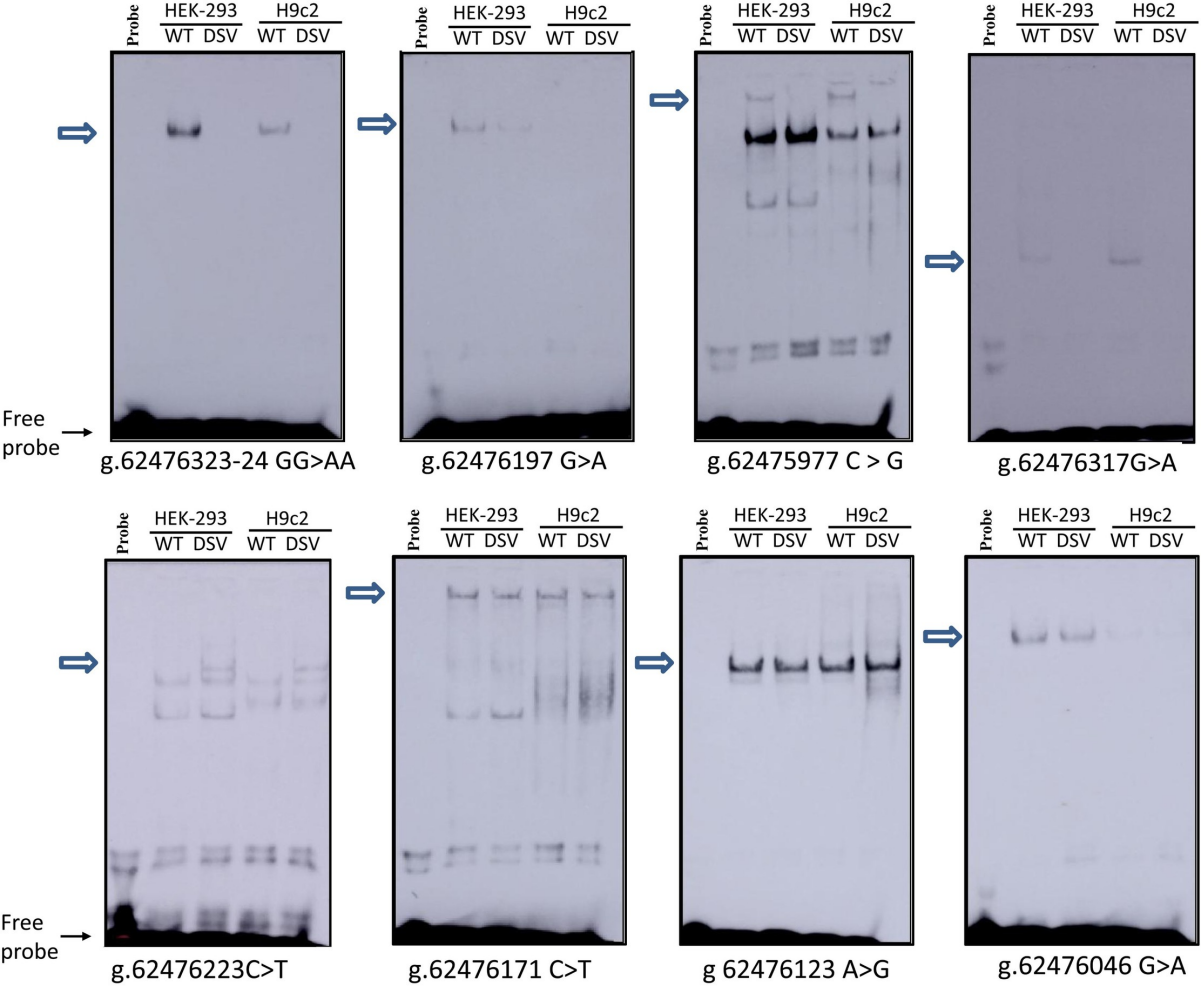
EMSA of biotin-labeled oligonucleotides containing DSVs. Wild-type and variant oligonucleotides (30 bp) were designed and labeled with biotin for the DSVs identified in AMI patients, including g.62476323-24GG>AA, g.62476197G>A, g.62475977C>G, g.62476317G>A, g.62476223C>T, g.62476171C>T, g.62476123A>G, and g.62476046G>A. EMSA was conducted with biotinylated oligonucleotides and the nuclear extracts from HEK-293 and H9c2 cells. Free probe was marked with an arrow at the bottom. The affected binding for transcription factors was marked with an open arrow.

Electrophoretic mobility shift assay (EMSA) was performed with variant and wild-type oligonucleotides (Table 1) to explore whether the DSVs interfered with the binding of TFs. In accordance with expectation, the DSV g.62476323-24GG>AA and the SNPs [g.62475977C>G (rs1294169077) and g.62476317G>A (rs80197101)] abolished the binding of a TF. The DSV g.62476197G>A markedly weakened the binding of a TF, but which was almost absent in H9c2 cells. The SNP g.62476223C>T (rs77067995) created a binding site for a TF. However, the DSV g.62476046G>A and the other two SNPs [g.62476171C>T (rs1341970027), g.62476123A>G (rs1435326263)] did not alter the binding of TFs, which might be due to the sensitivity of EMSA (Fig 5).

## 4. Discussion

Previous studies have shown that *GATA5* plays an important role in CVDs as *GATA5* loss-of-function mutations are closely related to congenital heart disease, atrial fibrillation, hypertension [5–8]. GATA5 deficiency can decrease the activity of AMPK and up-regulate the expression of bone morphogenic protein-4 (BMP-4), intercellular cell adhesion molecule-1 (ICAM-1) and interleukin-6 (IL-6) [7]. AMPK can promote the regeneration of coronary artery and exert cardioprotective effect under stress conditions such as myocardial ischemia/hypoxia and ischemia/reperfusion [14, 16, 17, 33]. However, the functional defect of GATA5 will weaken the protective effect of AMPK on myocardial cells, and then affect the occurrence and development of CVDs such as heart failure and AMI. BMP-4 represents the earliest measurable marker of atherosclerosis and is considered to be a mechanically sensitive autocrine cytokine, which plays an important role in promoting inflammation, hypertension and atherosclerosis [34]. When GATA5 deficiency [7], or vascular endothelial cells were stimulated by unstable blood flow [34], the production of BMP4 will increase. In addition, the functional defect of GATA5 can aggravate oxidative stress and endothelial dysfunction by increasing the activity of ubiquitin-proteasome system, which is associated with instability of coronary and carotid plaques [18]. Thus, we can see that GATA5 dysfunction may increase the susceptibility to AMI.

Our previous researches have found that five mutation sites in *GATA4* gene promoter [35], two mutation sites in *GATA5* gene promoter [36], and two mutation sites in *GATA6* gene promoter were associated with congenital heart disease [37]. Three mutation sites in *GATA4* gene promoter [19], and two mutation sites in *GATA6* gene promoter were related to AMI [20]. Now, we explored the relationship between *GATA5* gene promoter and AMI for the first time. In this study, nine mutations have been found in *GATA5* gene promoter, eight of them evidently altered the transcriptional activity of the *GATA5* gene promoter, five of them disrupted the binding of TFs. For example, g.62475977C>G (identified only in AMI patients) can abolish the binding of FXR (a transcriptional regulator that plays crucial role in the regulation of bile acids, lipids and glucose [25]) to *GATA5* promoter by changing the binding site of FXR on *GATA5* promoter, thereby reducing the transcriptional activity of *GATA5* gene promoter, and eventually increase the susceptibility to AMI by reducing the transcription of *GATA5* gene.

After Restriction Fragment Length Polymorphisms (RFLPs) and Variable Number of Tandem Repeats (VNTRs), SNP is the third kind of human DNA genetic marker. In order to find the dangerous genotype, allele and haploid of AMI, we analyzed the correlation between AMI and SNPs [g.62476317G>A (rs80197101) and g.62476223C>T (rs77067995)] of *GATA5* gene promoter. It was found that the frequency distribution of genotypes and alleles of rs80197101 and rs77067995 locus was significantly different between AMI and control groups. As for the effect to result in AMI, both the GA genotype of rs80197101 and the CT genotype of rs77067995 were 2.263 times higher than that of wild-type genotype, the genotypes of rs80197101 with A allele (GA and AA) are 2.297 times higher than that without A allele (GG), the genotypes of rs77067995 with T allele (CT and TT) are also 2.297 times higher than that without T allele (CC). Rs80197101 and rs77067995 showed perfect linkage disequilibrium and the haplotype AT (across rs80197101 and rs77067995) exhibited significantly higher frequencies in AMI group than in control group. These results suggested that the genotype GA and allele A of rs80197101, the genotype CT and allele T of rs77067995 and the haplotype AT are all risk factors of AMI.

In summary, the molecular genetic analysis of *GATA5* gene promoter was carried out, nine mutations have been found in *GATA5* gene promoter and these DNA variants may increase the susceptibility to AMI as risk factors. But the mechanism remains to be verified in *vivo*. Besides, this study only focused on *GATA5*. *GATA4*, *GATA5* and *GATA6* belong to the same subfamily of GATA family, whether they have interaction and the mechanism of their interaction in the occurrence and development of AMI remains unclear, which needs further study.

## Supporting information

S1 Raw images (PDF)
