# Supplementary figures and images for: Molecular genetic study on GATA5 gene promoter in acute myocardial infarction
Source: PLoS One. 2021 Mar 8;16(3):e0248203. doi: 10.1371/journal.pone.0248203 (PMC7939267; doi:10.1371/journal.pone.0248203)

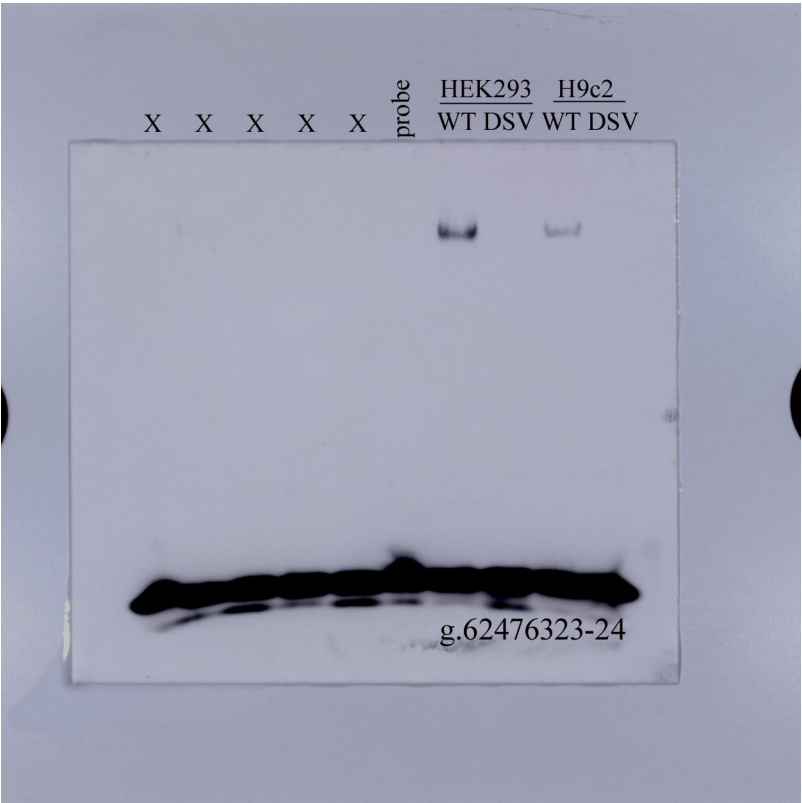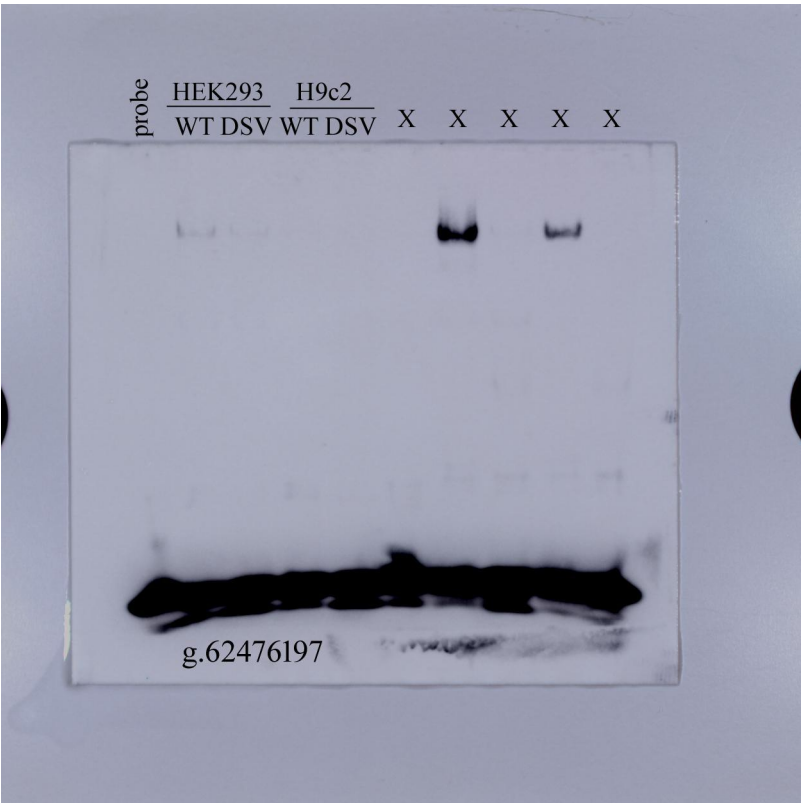

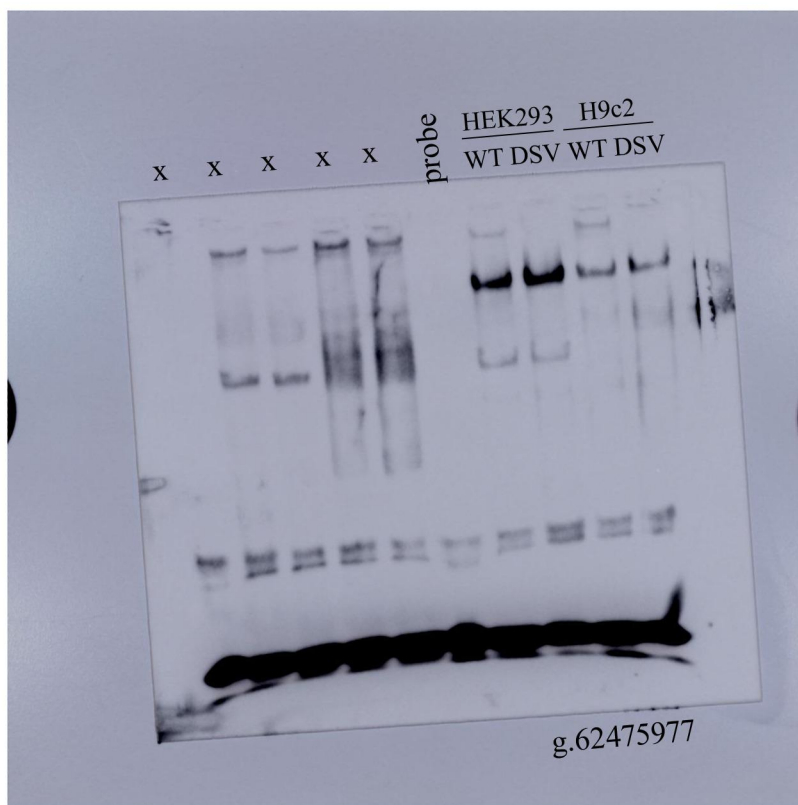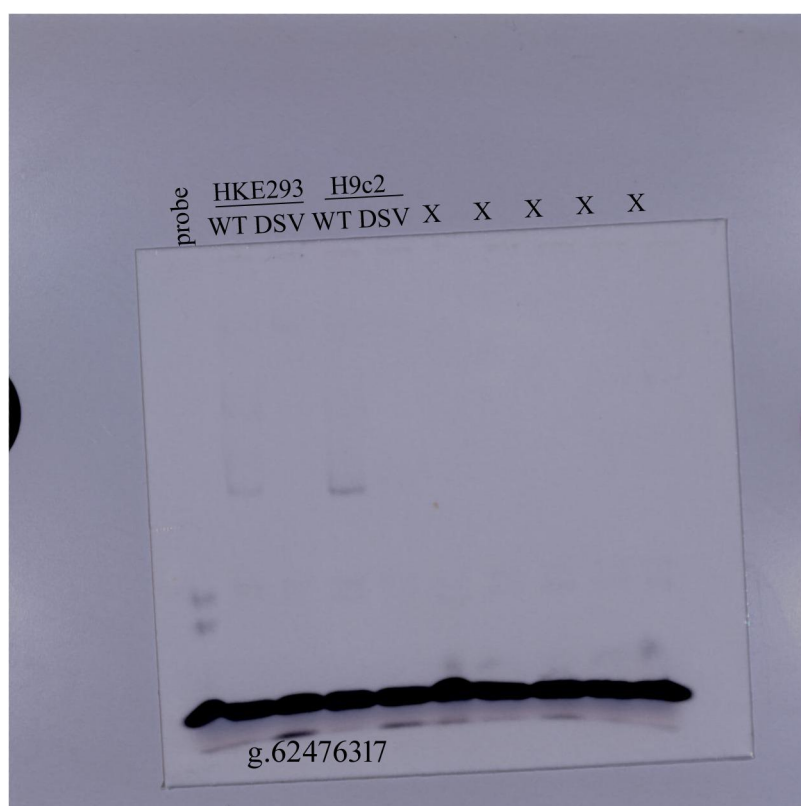

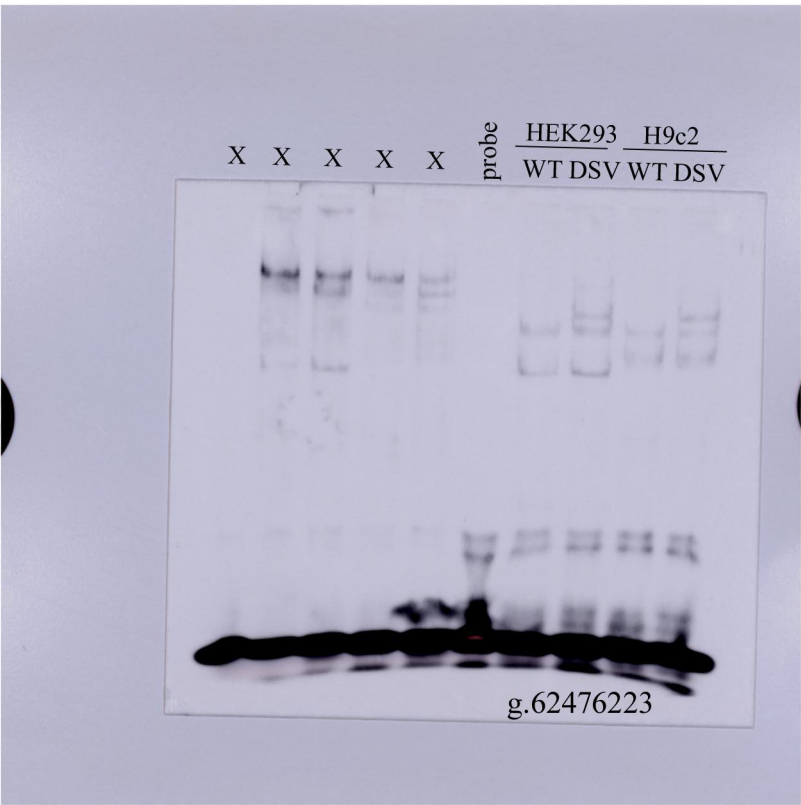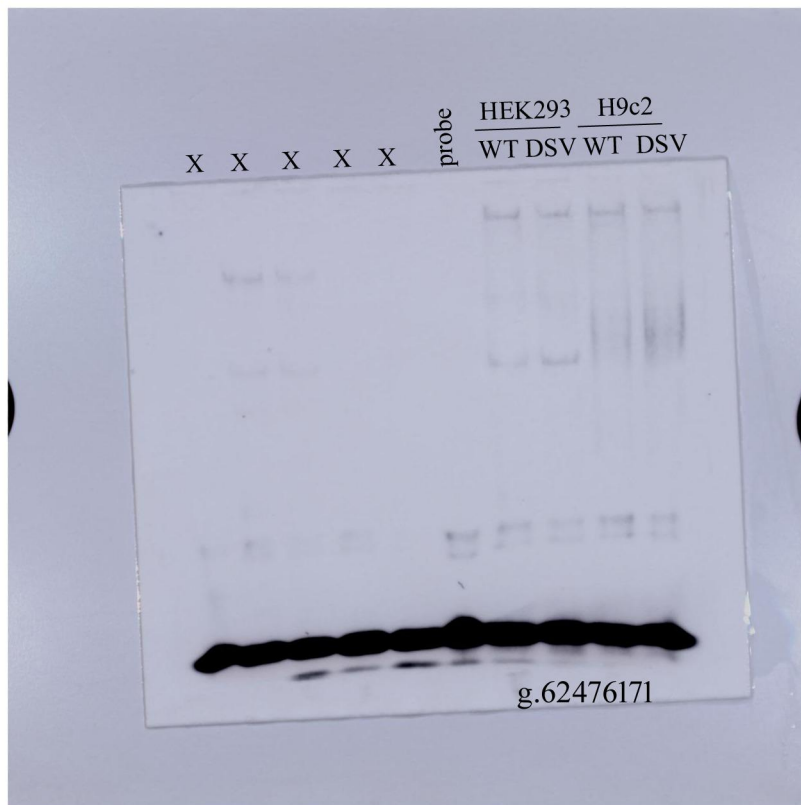

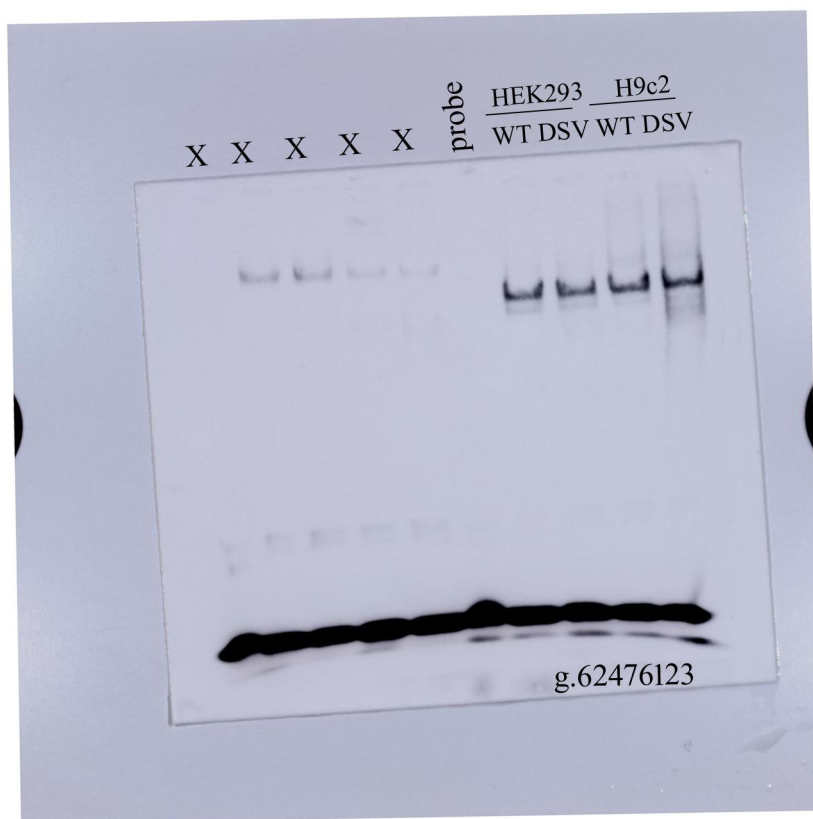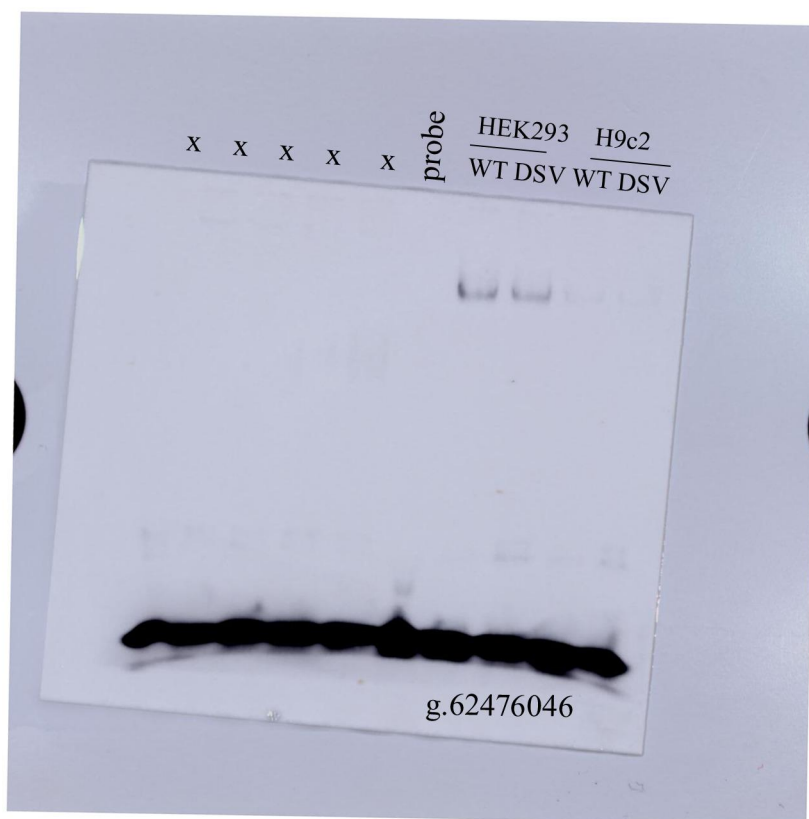

Supplement: S1 Raw images — (PDF) [file pone.0248203.s001.pdf]
